# Draft Genome Sequence of *Williamsia* sp. Strain D3, Isolated From the Darwin Mountains, Antarctica

**DOI:** 10.1128/genomeA.01230-13

**Published:** 2014-01-23

**Authors:** Leandro D. Guerrero, Thulani P. Makhalanyane, Jackie M. Aislabie, Don A. Cowan

**Affiliations:** a Centre for Microbial Ecology and Genomics, University of Pretoria, Pretoria, South Africa; b Landcare Research, Hamilton, New Zealand

## Abstract

Actinobacteria are the dominant taxa in Antarctic desert soils. Here, we describe the first draft genome of a member of the genus *Williamsia* (strain D3) isolated from Antarctic soil. The genome of this psychrotolerant bacterium may help to elucidate crucial survival mechanisms for organisms inhabiting cold desert soil systems.

## GENOME ANNOUNCEMENT

Antarctic soils include oligotrophic, copiotrophic, water-saturated, and hyperarid environments, but all are subject to extremely low seasonal temperatures (1). These conditions were thought to severely limit life, although recent phylogenetic surveys have demonstrated much higher than expected levels of prokaryote diversity from a range of terrestrial niches. Actinobacteria are ubiquitous soil inhabitants and a dominant component of Antarctic soils (2–4). *Williamsia* species have been isolated from a number of extreme environments (5, 6). To gain insight into the molecular mechanisms allowing survival under these extreme conditions, we have generated the first draft genome sequence of a novel psychrotrophic *Williamsia* sp.

*Williamsia* sp. strain D3 was isolated from soil from the Danum drift in the Lake Wellman area of the Darwin Mountains of Antarctica. The organism was isolated by plating soil onto R2A (Difco) agar plates and incubating at 15° C for up to 3 months (7). The genome of D3 was sequenced using an IonTorrent Sequencer (Life Technologies) at the DNA Sequencing Facility at the University of Pretoria. After quality filtering, 3,165,223 reads with an average size of 300 bp were assembled using MIRA v 4.0rc4 (8). The resulting contigs, with a minimum coverage of 45× and minimum length of 1,000 bp, were selected and joined manually, when possible, using GAP5 (9). The final draft comprised 50 contigs with a mean size of 112,462 bp and a maximum length of 501,473 bp. The total length of the genome was 5,623,123 bp with a mean GC content of 64.6% and an average coverage of approximately 145×. Annotation was performed by use of the NCBI Prokaryotic Genome Annotation Pipeline (http://www.ncbi.nlm.nih.gov/genome/annotation_prok). The draft genome contains 4,930 protein-coding sequences (3,475 with annotated functions and 1,455 hypothetical proteins), 45 tRNA genes, and 2 rRNA operons. However, the average contig coverage for the rRNA operons suggests that there may be up to three more copies. This is consistent with the number of rRNA copies estimated by the Ribosomal RNA Database (rrnDB) for the family (between 3 and 5 copies) (10). Based on comparisons of the 16S rRNA gene sequence, D3 was 100% identical with *Williamsia muralis*, the type species of the genus (11). Genes associated with cold stress were identified, including genes of the CspA family and an antifreeze protein of type I, which prevent ice formation.

The first draft genome sequence of this genus provides mechanistic insights into the metabolic adaptation of bacteria to cold environments as well to the characterization of the genetic potential of *Williamsia* sp. strain D3.

### Nucleotide sequence accession numbers.

This whole-genome shotgun project has been deposited at DDBJ/EMBL/GenBank under the accession number AYTE00000000. The version described in this paper is version AYTE01000000.
